# The Medical Research Committee and the War

**Published:** 1914-12-12

**Authors:** W. M. Fletcher

**Affiliations:** Secretary of the Medical Research Committee. St. Stephen's House, Westminster.


### THE EDITOR'S LETTER-BOX.

### The Medical Research Committee and the War.

*To the Editor of* The Hospital.

Sir,—In the article upon "Gas Gangrene," which appears on page 217 of your number of December 5, you speak of work which is being done at the Lister Institute, and conclude with the following words :—

"Incidentally the activities of the Lister Institute on these lines may be regarded as additional evidence, if such be needed, of the wisdom of its governors in their refusal to be absorbed by the Committee for Medical Research, established under the National Insurance Act. Institutions controlled by the Government are so much trammelled with their own inertia that they seldom achieve renown by their versatility and nimbleness of foot." I am desired to draw your attention to the serious errors of fact which are contained or implied in this paragraph.

In the first place, there has never been any question of the governors of the Lister Institute being absorbed by the Medical Research Committee. The governing body of the Institute, as is now well known to all readers of the chief medical journals, themselves originated the suggestion that the interests of the two bodies should be amalgamated. They proposed this amalgamation to the Medical Research Committee, and strongly recommended to the members of the Institute that the proposal should be adopted for the furtherance of the best interests of the Institute.

In the second place, it is not true that the Medical Research Committee are, as your words would seem to be intended to imply, so controlled by the Government as to be unable to bring their resources rapidly into effective action. The actual facts may be indicated shortly as follows.

Two days after the declaration of war the Committee placed their Central Institute building at Hampstead at the disposal of the War Office for use as a military hospital, and it is now being so used. From the outbreak of the war Sir Almroth Wright and other members of the scientific staff of the Committee have given their time at the inoculation department, St. Mary's Hospital, to prepare and supply to the War Office and to our Allies immense quantities of protective sera and vaccines, and probably more have been so supplied than by any other civilian organisation in the country.

At the present time the Committee are providing the services in France of Sir Almroth Wright and of two other members of their staff, and these are engaged in important researches, not only upon gas gangrene but upon other varieties of sepsis in wounds. The Committee have also sent a bacteriologist by way of Petrograd to Lemberg, where he is studying the particular strains of cholera there prevalent, so that in the possible contingency of a transference of German troops, and of that infection with them, to France, the best preventive measures may be immediately available for our troops upon this side.

By means of grants from the National Medical Research Fund, the Committee, with the sanction of the Army Council, have already arranged for special bacteriological work to be carried out at upwards of thirty of the largest hospitals for the wounded throughout the country, and this very extensive work, which is being almost daily increased, is being correlated as closely as possible with the experience gained by Colonel Sir William Leishman, a member of the Committee, and Sir Almroth Wright in the inquiries they are making at the Front.

Lastly, the Committee have undertaken, with the approval and by the authority of the Army Council, the heavy task of the compilation of the medical statistics of the war, and the statistical department of the Committee is already actively engaged upon this work.

In the initiation of all this varied emergency work in connection with the war, the Medical Research Committee, so far from being fettered by the Government, have found the most complete encouragement to use promptly, and to the fullest extent, the national resources entrusted to their direction, and to apply them with flexibility and effect to meet the present national needs. I ought to add that inquiries of the simplest kind might have prevented the appearance in your paper of the grave misrepresentations to which I have drawn attention.—I am, Sir, yours faithfully,

W. M. Fletcher,

Secretary of the Medical Research Committee.

St. Stephen's House, Westminster.

December 8, 1914.

[The preceding letter reaches us as we go to press, and it contains two obvious errors. First, the word "absorbed" to which objection is taken in the paragraph quoted is obviously used metaphorically, for a reference to our leading article on "The State Control of Research" in The Hospital of the previous week shows that the facts are fully within our knowledge. As to point 2 : The last sentence of the paragraph quoted remains, what it professed to be, generally correct as a rule of experience. No one, however, can be more delighted than ourselves to learn that the Medical Research Committee is one of the exceptions, the existence of which our use of the word "seldom" implied.—Ed. The Hospital.]

